# Electroluminescence from pure resonant states in hBN-based vertical tunneling junctions

**DOI:** 10.1038/s41377-024-01491-5

**Published:** 2024-07-08

**Authors:** Magdalena Grzeszczyk, Kristina Vaklinova, Kenji Watanabe, Takashi Taniguchi, Konstantin S. Novoselov, Maciej Koperski

**Affiliations:** 1 Institute for Functional Intelligent Materials, National University of Singapore, Singapore, 117544 Singapore; 2 Research Center for Functional Materials, National Institute for Materials Science, Tsukuba, 305-0044 Japan; 3 International Center for Materials Nanoarchitectonics, National Institute for Materials Science, Tsukuba, 305-0044 Japan; 4 Department of Materials Science and Engineering, National University of Singapore, Singapore, 117575 Singapore

**Keywords:** Lasers, LEDs and light sources, Electronics, photonics and device physics, Optical materials and structures, Single photons and quantum effects

## Abstract

Defect centers in wide-band-gap crystals have garnered interest for their potential in applications among optoelectronic and sensor technologies. However, defects embedded in highly insulating crystals, like diamond, silicon carbide, or aluminum oxide, have been notoriously difficult to excite electrically due to their large internal resistance. To address this challenge, we realized a new paradigm of exciting defects in vertical tunneling junctions based on carbon centers in hexagonal boron nitride (hBN). The rational design of the devices via van der Waals technology enabled us to raise and control optical processes related to defect-to-band and intradefect electroluminescence. The fundamental understanding of the tunneling events was based on the transfer of the electronic wave function amplitude between resonant defect states in hBN to the metallic state in graphene, which leads to dramatic changes in the characteristics of electrons due to different band structures of constituent materials. In our devices, the decay of electrons via tunneling pathways competed with radiative recombination, resulting in an unprecedented degree of tuneability of carrier dynamics due to the significant sensitivity of the characteristic tunneling times on the thickness and structure of the barrier. This enabled us to achieve a high-efficiency electrical excitation of intradefect transitions, exceeding by several orders of magnitude the efficiency of optical excitation in the sub-band-gap regime. This work represents a significant advancement towards a universal and scalable platform for electrically driven devices utilizing defect centers in wide-band-gap crystals with properties modulated via activation of different tunneling mechanisms at a level of device engineering.

## Introduction

The functionalities of crystals at the atomic scale can be activated by the systematic, controllable, and precise formation of defect centers. From this perspective, wide-band-gap materials are particularly attractive^1–3^, as the midgap defect levels are often decoupled from the fundamental electronic bands, consequently becoming pure quantum systems^4–7^. When properly controlled, defect centers act as qubits^8,9^, single photon emitters^10–15^, sensors of pressure^16,17^, magnetic fields^18–20^, electric fields^21^, thermal conductivity^22^, or dielectric constant^23^ at the ultimate limit of miniaturization. They also activate the optical response of materials, enabling the realization of lasers^24^ and photodetectors^25^. Defect levels located deep within the band gap^26^ enable room temperature operation of devices, making research in such centers highly technological. However, all of these functionalities suffer from a significant bottleneck resulting from the insulating character of the host crystals. Large internal resistance prevents, in most cases, the development of electrically driven devices^27–29^. Notably, in rare cases when the n-type and p-type doping of the insulators can be controllably and efficiently introduced, intradefect electroluminescence (EL) could be realized in three-dimensional materials^30,31^ through the creation of p–n junctions.

In this study, we explored an alternative path toward electrical excitation of defect centers realized with thin films of hexagonal boron nitride (hBN) integrated into van der Waals (vdW) vertical tunneling junctions. Conceptually, the design of the devices was adapted from the semiconductor technology^32^, however, their operation was based on fundamentally different principles. The key enabler of the transition from semiconducting to insulating defect-based light-emitting diodes (LEDs) came from realizing specific radiative centers in hBN through carbon-doping^33^. The emerging defects led to homogeneous photoluminescence (PL) signals resembling the optical response of pristine excitonic semiconductors rather than disordered defect-based systems. This allowed us to design devices where the defect levels acted as purely resonant states, providing dominant tunneling pathways between two graphene (Gr) electrodes. As the timescales of the tunneling processes are strongly dependent on the barrier width and structure, we demonstrated a transition from defect-to-band to intradefect EL and electron-to-photon conversion modulated by almost two orders of magnitude via rational engineering of the device architecture.

Our findings are relevant for the advancements in insulating defect-based LEDs, providing general guidelines for the transition from p–n junction to vertical tunneling junction geometries. The latter structures can achieve significant levels of tunability of the optoelectronic properties of devices based on the understanding of the tunneling processes mediated by resonant defect states.

## Results

### Samples

**Fig. 1:**
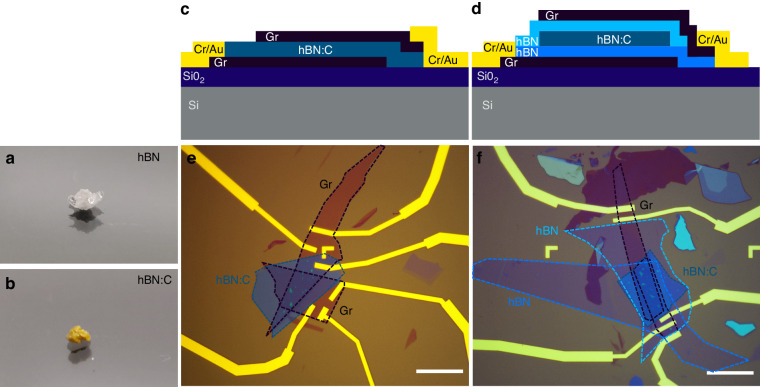
The architecture of the vertical tunneling devices with hBN:C as an energy barrier. The optical images of the hBN (**a**) and hBN:C bulk crystals (**b**). Schematic representation of the light emitting diode structures with the layer sequence Gr/hBN:C/Gr (**c**) and Gr/hBN/hBN:C/hBN/Gr (**d**). The devices were created by stacking mechanically exfoliated graphene, hBN, and hBN:C layers. The optical images of example devices with Gr/hBN:C/Gr and Gr/hBN/hBN:C/hBN/Gr architectures are presented in (**e**) and (**f**), respectively. The thickness of hBN:C was ~20 nm, while the hBN barriers were ~3–5 nm thick. The scale bar corresponds to 10 μm

The creation of hBN-based LEDs was enabled through the introduction of well-defined, homogeneously distributed, and reproducible radiative defect centers through carbon doping^34^. The crystallographic quality of hBN must be high for thin films to fulfill the role of a tunneling barrier^35^. To that end, we grew the bulk hBN crystals via high pressure, temperature-gradient method^36^. From a single growth, we annealed a fraction of the crystals in a graphite furnace at the temperature of 2000 °C for a period of 1–5 h to achieve varied concentrations of carbon impurities. The carbon-doped hBN (hBN:C) became yellow to blackish, depending on the annealing time, contrary to the pristine transparent hBN (see Fig.1a, b for comparison of the optical images between bulk hBN and hBN:C crystals).

The carbon doping procedure activated multiple resonances in the PL spectra in the ultraviolet, visible, and near-infrared spectral regions^33,37,38^. The translation from optical to electrical excitation requires understanding and controlling the dynamics of charge carriers participating in the tunneling and radiative recombination processes. Therefore, we have fabricated two types of devices with different architectures of the tunneling barrier, which are schematically depicted in Fig. 1c, d. A Si/(290 nm) SiO_2_ wafer was used as the substrate for the vdW heterostructure with the following layer sequence: (1) Gr/hBN:C/Gr and (2) Gr/hBN/hBN:C/hBN/Gr. The optically active hBN:C layer was ~20 nm thick and the additional hBN spacer was 3–5 nm thick for the devices discussed herein. Optical microscope images of the representatives of the two classes of devices are shown in Fig. 1e, f. Details about device fabrication can be found in the “Materials and methods” section.

**Fig. 2:**
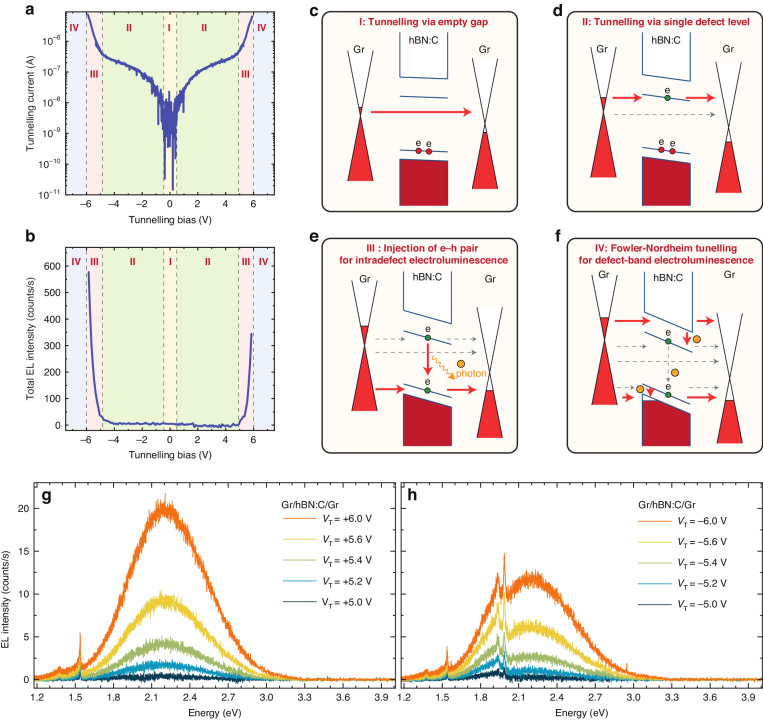
Electroluminescence from Gr/hBN:C/Gr devices. Tunneling *IV* curve for a Gr/hBN:C/Gr (**a**) demonstrates activation of novel tunneling paths. The device displays electroluminescence at a higher bias threshold, which can be seen from the dependence of the integrated light emission intensity on the tunneling bias (**b**). The integration is done in the spectral region 1.2–3.9 eV. Schematic depiction of the tunneling processes based on the band structure of the heterostructure and the Fermi level alignment with bias is presented in various regimes: tunneling via an empty gap for small bias voltage (**c**), tunneling via a single defect level with an increased bias voltage (**d**), formation of electron–hole pairs activating intradefect electroluminescence (**e**), defect-to-band electroluminescence in the sup-band-gap bias regime (**f**). The electroluminescence spectra for different bias voltages are presented under the forward (**g**) and reverse (**h**) biasing direction. All measurements were made at *T* = 5 K

We begin with the characterization of the Gr/hBN:C/Gr device, whose *IV* characteristics are demonstrated in Fig. 2a. The zero-bias resistance yielded 0.1 GΩ, which indicated that the direct Gr–Gr tunneling through the barrier defined by the hBN band gap, illustrated schematically in Fig. 2c, was inefficient. The conductance related to such a tunneling process^39^ is given by $G\propto \exp$($\frac{-2d\sqrt{2m* {hBN}\phi B}}{\hslash })$, where *d* is the thickness of the tunneling barrier, m^*^_hBN_ is the effective mass of an electron in hBN, $\phi B$ is the barrier height and $\hslash$ is the reduced Planck’s constant. The utilization of a relatively thick hBN barrier (20 nm) characterized by a 6 eV band gap enabled us to quench the direct Gr–Gr tunneling mechanism. The first onset of tunneling was observed at the bias of about ±0.5 V, which was associated with the threshold related to the resonant alignment of the Fermi level in graphene with a defect state energy in hBN as shown in Fig. 2d. This constitutes a qualitatively different tunneling mechanism driven by the transfer of the amplitude of the electron’s wave function between the metallic state in graphene to the resonant defect state in the hBN barrier. This process resembles tunneling through a bound resonant state in a semiconductor quantum well^40^ with the characteristic tunneling time given by *τ*_tunnel_ = *τ*_0_$\,\exp (\frac{2d\sqrt{2m* (e\phi B-{E_{{\rm {res}}}})}}{\hslash })$, where *τ*_0_ is a normalization factor, m* is the effective mass of the electron in the quantum well, *e* is the elementary charge, *E*_res_ is the energy of the resonant state. *τ*_tunnel_ is indicative of the decay of the wave function amplitude from the resonant defect state and becomes the key factor in controlling the optoelectronic properties of the tunneling junctions. In the Gr/hBN:C/Gr device, the interpretation of *τ*_tunnel_ is more intricate, as the tunneling process implies the gradual transformation of the characteristics of an electron from a massless particle in graphene to a resonant dispersionless defect state in hBN. Such a process cannot be captured by the effective mass approximation, therefore it is challenging to make quantitative predictions for *τ*_tunnel_ in different device architectures. In typical semiconductor tunneling junctions, the contribution of the resonant state to the wave function of the electron remains very small throughout the entire tunneling event due to lower barrier heights^40^. In contrast, the defect states in hBN exhibited purely resonant characteristics, evidenced by the negligible current due to direct Gr–Gr tunneling at the onset of the activation of defect-mediated tunneling. The resonant characteristics are strengthened in hBN due to the large value of the band gap compared with the low onset of defect-mediated tunneling at about 0.5 V, which demonstrated that the defect levels are decoupled from the electronic bands. Moreover, the wave functions of electrons occupying the defect level in hBN are typically localized within volumes characterized by dimensions below several nanometers^37^, which facilitates their confinement in the sizable barriers. In the regime marked by the first tunneling onset, EL was not allowed due to the absence of an empty final state of the recombination process if we assume the more likely case of tunneling via a donor-like defect level below the conduction band. A second tunneling onset related to the injection of the hole into an acceptor-like defect level (see Fig. 2e) was observed at 5 V, which coincided with the detection of light emitted by the device.

## Discussion

The inspection of the EL spectra, which are demonstrated in Fig. 2g, h, granted further insight into the competition between the tunneling processes and radiative recombination. Two types of qualitatively different contributions to the EL spectra could be identified: (1) broadband features with a width of about 1 eV, which could be attributed to transitions between the mid-gap defect level and electronic sub-bands^41^ and (2) narrow resonances characteristic of intradefect excitations. The activation of the defect-to-band EL requires a different tunneling process via the Fowler–Nordheim mechanism^42,43^. Either an electron can be injected into the conduction band (Fig. 2f), recombining with the underlying empty defect level, or a hole can be injected into the valence band, enabling the recombination of an electron occupying a defect level. In general terms, the bias threshold for the defect-to-band and intradefect optical transitions is determined by the electronic structure of the mid-gap levels in combination with the position of the Fermi level in graphene in relation to the band edges of hBN forming a triangular tunneling barrier. In the Gr/hBN:C/Gr device, the threshold for both types of EL coincided at 5 V when the tunneling current was 400 nA. The low contribution from the intradefect transitions to the EL spectra indicated, that the Gr/hBN:C/Gr device operated in the regime *τ*_tunnel_ « *τ*_rad_, where *τ*_rad_ is the radiative lifetime of the electron determining the decay of photoluminescence intensity *I*_PL_(*t*) = *I*_0_ $\exp (-t/$*τ*_rad_*)*, where *I*_0_ is a normalization constant, *t* is a time typically measured with respect to a short laser pulse, and *τ*_rad_ is the radiative lifetime. *τ*_rad_ was previously measured to be 1.0 ± 0.1 ns for the intradefect transition at 1.54 eV and 1.3 ± 0.2 ns for the intradefect transition at 1.99 eV in hBN:C films^33^. These values act as an estimation of the upper limit for *τ*_tunnel_ in the Gr/hBN:C/Gr devices.

**Fig. 3:**
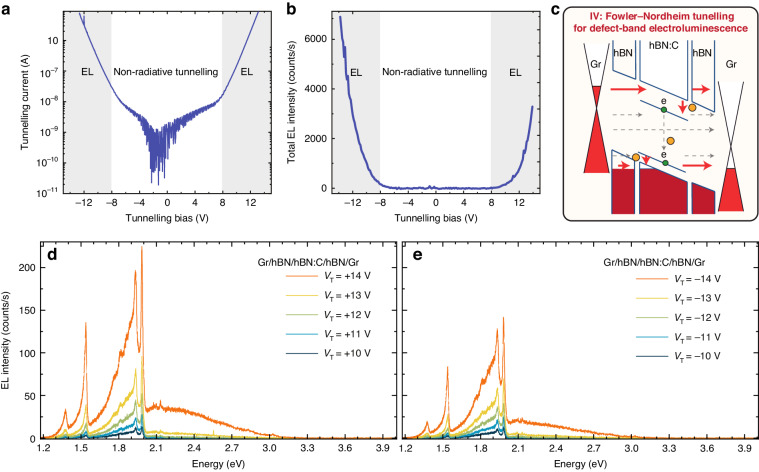
Electroluminescence from Gr/hBN/hBN:C/hBN/Gr devices. Tunneling *IV* curves (**a**) and the integrated EL intensity dependence on the tunneling bias (**b**) demonstrate the increase of the tunneling and electroluminescence voltage threshold induced by additional pristine hBN barriers. Schematic depiction of the charge tunneling processes in the sup-band-gap voltage regime enabling non-radiative Gr–Gr tunneling (including tunneling mediated by electronic bands via Fowler–Nordheim mechanism), defect-to-band electroluminescence and intradefect electroluminescence (**c**). The evolution of the electroluminescence spectra with bias voltage under forward (**d)** and reverse (**e**) direction demonstrates that the electroluminescence spectra are dominated by intradefect optical transitions. All the measurements were done at *T* = 5 K

In order to access *τ*_tunnel_ » *τ*_rad_ regime favoring the efficiency of intradefect radiative transitions, we fabricated Gr/hBN/hBN:C/hBN/Gr devices with enhanced barrier width and additional non-aligned hBN/hBN:C interfaces. The zero-bias resistance of these devices increased by more than two orders of magnitude to the value of ~20 GΩ. The sequential tunneling onsets were not visible in the *IV* curves due to the large resistance (Fig. 3a). In such a device, the onset of EL was driven by the current rather than the voltage threshold. The light emission appeared at the bias of 8 V, which corresponded to the tunneling current of 10 nA (Fig. 3b). Although the voltage threshold increased, the EL required a 40-times smaller current to be activated. The reduction in the threshold tunneling current coincided with a qualitative modification of the EL spectra, as demonstrated in Fig. 3d, e. In this device architecture, the intradefect transitions characterized by narrow linewidth resonances dominated the broadband contribution associated with defect-to-band transitions. The total emission intensity, integrated over the spectral region 1.2–3.3 eV increased significantly. To provide a rational comparison, the integrated EL signal normalized by the area of the device was about 80 times stronger for the Gr/hBN/hBN:C/hBN/Gr device than for Gr/hBN:C/Gr device for the same current of 1 μA, as can be seen in Fig. S11.

We interpret the barrier-induced modifications of the optoelectronic properties of our tunneling devices in terms of the band alignment schematically illustrated in Fig. 3c. The enhanced efficiency of EL originated from the increase in *τ*_tunnel_ affected by the introduction of additional pristine hBN barriers. Concurrently, the operation of the Gr/hBN/hBN:C/hBN/Gr device in the Fowler–Nordheim tunneling regime led to quenching of the efficiency of the defect-to-band transition, as the direct Gr–Gr tunneling directly through the conduction and/or valence band became a dominant tunneling mechanism for electrons occupying states close to the Fermi levels in graphene electrodes due to the reduction of the effective barrier width with increasing voltage in the presence of a triangular tunneling barrier. Notably, our devices often exhibited an asymmetry of the *IV* characteristics and the EL intensity dependence on the tunneling bias. The origin of the asymmetry could be attributed to a variation in the number of hBN layers and/or the varied quality of the interfaces determined by the stacking process, as the symmetric design of our structures implies that the charge carrier dynamics should not depend on the sign of the applied bias. From the practical perspective, our devices exhibited remarkable stability, as demonstrated by the consistent emission in terms of intensity and energy of the optical resonances tested over a 10-minute duration, as shown in the Supplementary Information (SI) in Fig. S3. Furthermore, we conducted temperature-dependent measurements, revealing that the emission remained distinguishable with characteristic resonances even at room temperature, underlining the versatility of these structures for practical applications. The temperature-dependent EL spectra are presented in SI in Fig. S4.

**Fig. 4:**
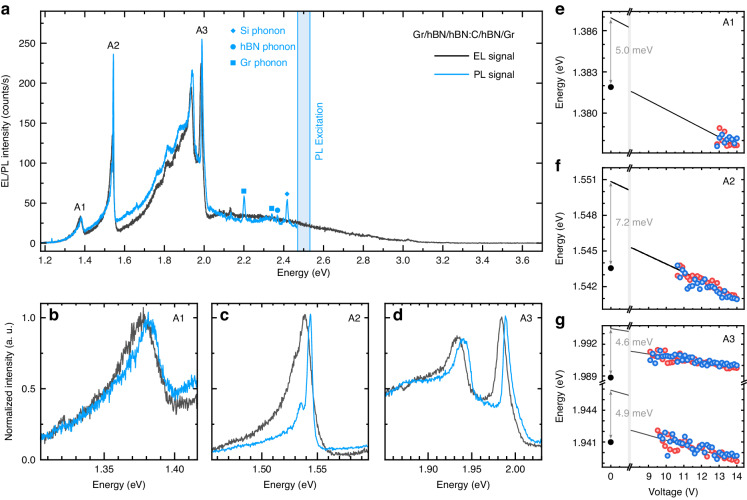
Electrical and optical excitation of carbon centers in hBN and the bias-driven control of the emission energy. The comparison of the photoluminescence and electroluminescence spectra for the Gr/hBN/hBN:C/hBN/Gr device (**a**). The photoluminescence spectrum was collected with 2.56 eV laser excitation. The laser was focused to a spot of 1 μm diameter with a power of 1 mW. Electroluminescence was measured with −14 V bias voltage, which corresponded to *I*_T_ = −2 μA. Photoluminescence and electroluminescence spectra were collected via the same microscope objective from a region of the sample with a size close to the diffraction limit for the adequate comparison of emission intensity. All individual emission centers, A1 (**b**), A2 (**c**), and A3 (**d**), are present in both optically and electrically excited spectra. The attribution to a specific defect complex of each emission resonance was previously reported in ref. ^38^. The energy of emission for defects A1 (**e**), A2 (**f**), and A3 (**g**) changes with the tunneling bias in forward (red dots) and reverse (blue dots) directions. The extrapolated zero-bias energy is offset by a few meV from the energy of the resonance in the photoluminescence spectrum (black dot in **e**–**g**). All measurements were made at *T* = 5 K

Our work provides general guidelines that can be universally applied to transition from understanding the physical mechanisms of the tunneling processes to the activation/quenching of specific radiative pathways and modulation of the light emission efficiency of optoelectronic devices. In order to provide a quantitative context for this analysis, we compared the PL and EL spectra from a single device under identical physical conditions, which are presented in Fig. 4a. The presence of multiple resonances near the excitation energy in the PL spectra, notably absent in the EL response, originated from phonon modes associated with the constituent materials of the device (Si, Gr, and hBN). We identified three types of defects contributing identical signatures of intradefect resonances to PL and EL spectra, which we labeled A1–A3. Their origin was related to intradefect transitions characterized by varied degrees of electron–phonon coupling quantified by Huang–Rhys factors, as thoroughly discussed in ref. ^38^. A comparable emission intensity of all three resonances was observed for optical excitation at 2.56 eV with the power of 1 mW and for the electrical excitation with the current of 2 μA. These values correspond to excitations with 2.4 × 10^15^ photons per second and 1.2 × 10^13^ electrons per second, which was further reduced to 2.5 × 10^10^ electrons per second if we consider tunneling only through the area from which we collect light in the confocal geometry. Such interpolation was justified by the homogenous EL signal, as illustrated in Fig. S2. These considerations imply that the photon-to-photon conversion was about five orders of magnitude smaller than the electron-to-photon conversion. The significantly enhanced quantum efficiency for electrical excitation can be understood from geometrical and band structure considerations. The optical excitation was realized in the sub-band-gap regime, in which photons are absorbed exclusively at the defect sites in resonant conditions^33^ (i.e., the host crystal is optically transparent). The *IV* characteristics of our devices demonstrated that the tunneling current in the sub-band-gap bias regime in the absence of available defect states was negligible (i.e., the host crystal is not electrically conductive). Hence, in a simple view, the hBN crystal is transparent for photons but opaque for electrons in the mid-gap regime.

The realization of the electrical excitation enabled control of the energy of the optical resonances, as illustrated in Fig. 4b–d. In the Fowler–Nordheim tunneling regime, we observed a redshift of the emission energy for defects A1–A3 upon increasing the tunneling bias. We attributed this observation to an effect of screening by electrons injected into the conduction band of hBN. The increase in effective dielectric constant leads to a reduction in the energy of the intradefect optical transitions related to carbon and vacancy centers in hBN^38^. In the mid-gap regime for the Gr/hBN:C/Gr devices, the evolution of the emission energy was qualitatively different, in agreement with a Stark effect^44,45^, as illustrated in Fig. S15. Therefore, the energy tunability in our devices was driven by a competition between the dielectric screening and the Stark effect, with the dominant contribution determined by the tunneling regime. While single-state considerations are sufficient to account for experimental observations, we expanded our discussion to include many-body states in SI (see Fig. S16).

In conclusion, we have successfully developed LEDs based on vertical tunneling junctions utilizing hBN:C as the barrier material. We have achieved a unique regime where mid-gap defect levels acted as resonant states, resulting in highly tunable electron tunneling mechanisms. Through optoelectronic characterization of these devices, we have uncovered universal principles connecting the electrical performance of the device with its optical response, governed by electron dynamics controllable via band structure engineering. Despite the technological challenges posed by LEDs based on insulating materials, they offer access to purely resonant states and unprecedented control over the efficiency of radiative transitions, owing to high tunneling barriers. Our findings pave the way for optoelectronic applications leveraging wide-band-gap 2D materials, which have the potential to fully exploit the advantages of miniaturized tunneling devices.

## Materials and methods

### Sample fabrication

Graphite, hBN, and hBN:C crystals were mechanically cleaved onto silicon wafers with 300 and 90 nm layers of SiO_2_, respectively. Thin graphite films were selected to act as electrodes for hBN:C of ~20 nm thickness, identified by optical contrast and atomic force microscopy.

Using a polydimethylsiloxane/polycarbonate stamp, the assembly of graphite/hBN/hBN:C/hBN or graphite/hBN:C/ was lifted from the Si/ SiO_2_ wafer at 100 °C. Subsequently, the stacks were released onto the pre‐exfoliated graphite flake together with the polycarbonate film at 180 °C, which was washed away thereafter by using dichloromethane, acetone, and isopropanol to remove polymer residues. The electrical contacts to the top and bottom graphite electrodes were patterned using electron beam lithography followed by evaporation of 5 nm‐Cr/60 nm‐Au layer and finalized by a lift‐off process.

### Experimental setup

The optical spectra were measured in dry cryogenic systems with a base temperature of 4.2 K. The sample was cooled down via thermal contact with a cold finger. The laser light was focalized on the surface of the device, and the PL/EL signal was collected through an in‐situ objective with a numerical aperture of 0.82. The sample was mounted on a chip carrier positioned on a set of *x*/*y* piezo‐positioners that allow alignment, while the objective was fixed on a piezo positioner *z* that allows focalization of laser light. The optical signal was dispersed by a 0.75 m spectrometer equipped with a 150 g/mm grating. The light was detected by a liquid nitrogen-cooled charge-coupled device camera. For PL excitation *λ* = 488 nm (2.56 eV) or *λ* = 514 nm (2.41 eV) continuous wave (CW) laser diode was used. The excitation power focused on the sample was kept at 1 mW. The current–voltage characteristics were measured with a Keithley 24XX source meter.

### Supplementary information

Supplementary Information for: Electroluminescence from Pure Resonant States in hBN-based Vertical Tunnelling Junctions contains temperature-dependent EL, stability of the device performance, a summary of other studied devices and their performance, additional data on dielectric breakdown and its effect on the EL, sample homogeneity, recombination pathways for defect-to-band emission, more detailed description of the emission behavior versus applied bias voltage, and description of an alternative many-body picture of charging and EL mechanism.

## Data Availability

The data that support the findings of this study are openly available at the following 10.58132/B4QQ5E.
