# Supplementary material for: Electroluminescence from pure resonant states in hBN-based vertical tunneling junctions
Source: Light Sci Appl. 2024 Jul 8;13:155. doi: 10.1038/s41377-024-01491-5 (PMC11231135; doi:10.1038/s41377-024-01491-5)
Supplement: Supplementary file 1 — Supplementary Information for: Electroluminescence from Pure Resonant States in hBN-based Vertical Tunnelling Junctions contains temperature-dependent EL, stability of the device performance, a summary of other studied devices and their performance, additional data on dielectric breakdown and its effect on the EL, sample homogeneity, recombination pathways for defect-to-band emission, more detailed description of the emission behavior versus applied bias voltage, and description of an alternative many-body picture of charging and EL mechanism. [file 41377_2024_1491_MOESM1_ESM.pdf]

Supplementary Information for: Electroluminescence from Pure Resonant States in hBN-based Vertical Tunnelling Junctions.

M. Grzeszczyk,*,† K. Vaklinova,† K. Watanabe,‡ T. Taniguchi,¶
K. S. Novoselov,†,§ and M. Koperski*,†,§

†*Institute for Functional Intelligent Materials, National University of Singapore, 117544, Singapore*

‡*Research Center for Functional Materials, National Institute for Materials Science, Tsukuba 305-0044, Japan*

¶*International Center for Materials Nanoarchitectonics, National Institute for Materials Science, 305-0044, Japan*

§*Department of Materials Science and Engineering, National University of Singapore, 117575, Singapore*

E-mail: magda@nus.edu.sg; msemaci@nus.edu.sg

Homogeneity assessment via Raman Scattering mapping

We conducted low-temperature (5 K) Raman scattering mapping on a representative sample with Gr/hBN/hBN:C/hBN/Gr architecture. In Fig. S1 we demonstrate a vibrational signature throughout the area, highlighting the uniformity and quality of our samples. The phonon peak

at energy $\sim$ 1375 cm^{-1} correspond to E_{2g} vibration in hBN and can be observed throughout the map region due to the presence of the pristine hBN barrier. Its intensity decreases at the active sample side as the Gr layer is placed on top of the structure. In contrast, signal coming from Gr contacts, mainly G– and 2D–band, at energies around 1589 cm^{-1} and 2710 cm^{-1}, respectively exhibit the highest intensity in the overlap area.

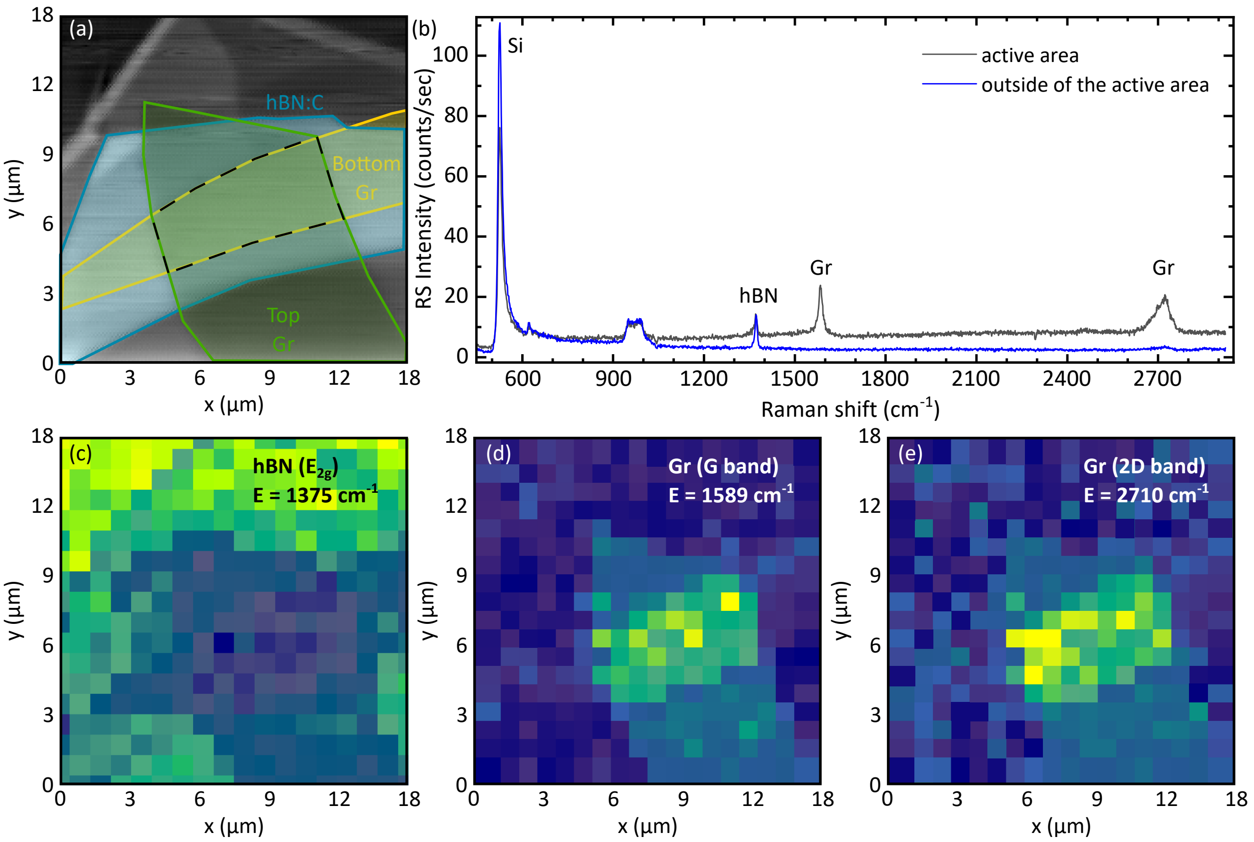

Figure S1: (a) Optical image of the sample with indicated layers. Black dashed line demarcate the active region of the device. (b) Representative Raman spectra obtained from within the active area (black curve) and outside the active area (blue curve) showcasing characteristic peaks corresponding to different layers within the sample. Intensity mapping of specified phonon peaks in the Raman scattering spectra, highlighting variations in intensity across the sample for (c) hBN flakes, (d) G band, and (e) 2D band coming from graphite contacts. Sample label B04.

Homogeneity assessment via electroluminescence mapping

Fig. S2(a) shows an optical image of the Gr/hBN:C/Gr sample, with a designated area that was scanned, highlighted by a white square. A corresponding electroluminescence map at low temperature (5 K) of the sample is displayed in Fig. S2(b) when a bias voltage of -7 V was applied. The same set of data is presented for Gr/hBN/hBN:C/hBN/Gr, with the optical image presented in Fig. S2(c) and the electroluminescence map shown in Fig. S2(d) when a bias voltage of -12.5 V was applied. In both cases, the entire area emits light in a very similar manner, revealing that the samples are remarkably uniform in their electroluminescent properties.

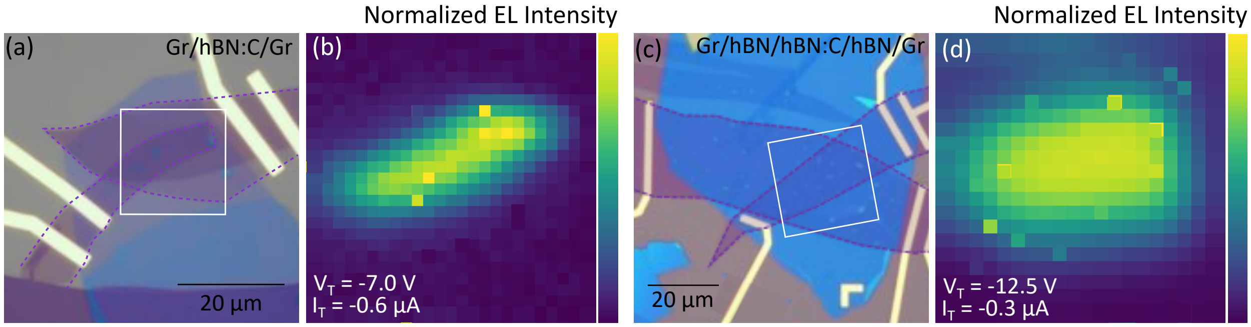

Figure S2: Optical microscope images of the representative devices for both examined architectures (a) Gr/hBN:C/Gr and (c) Gr/hBN/hBN:C/hBN/Gr. Scanning electroluminescence images measured at bias V_T of -7.0 V (b) and V_T of -12.5 V (c). A homogenous signal at energy $\approx$ 2 eV is observed where the hBN:C overlaps with the graphene electrodes.

Temporal stability of devices

The stability of electroluminescent devices is a critical factor in their practical applicability, particularly in optoelectronics and sensing. To assess their reliability, we performed extensive measurements at low temperature (5 K) over a duration of ten minutes and monitored the emission characteristics. These measurements aimed to reveal any fluctuations or degradation of the electroluminescence signal, which could be detrimental for various applications. Remarkably, the results showed exceptional temporal stability in our devices.

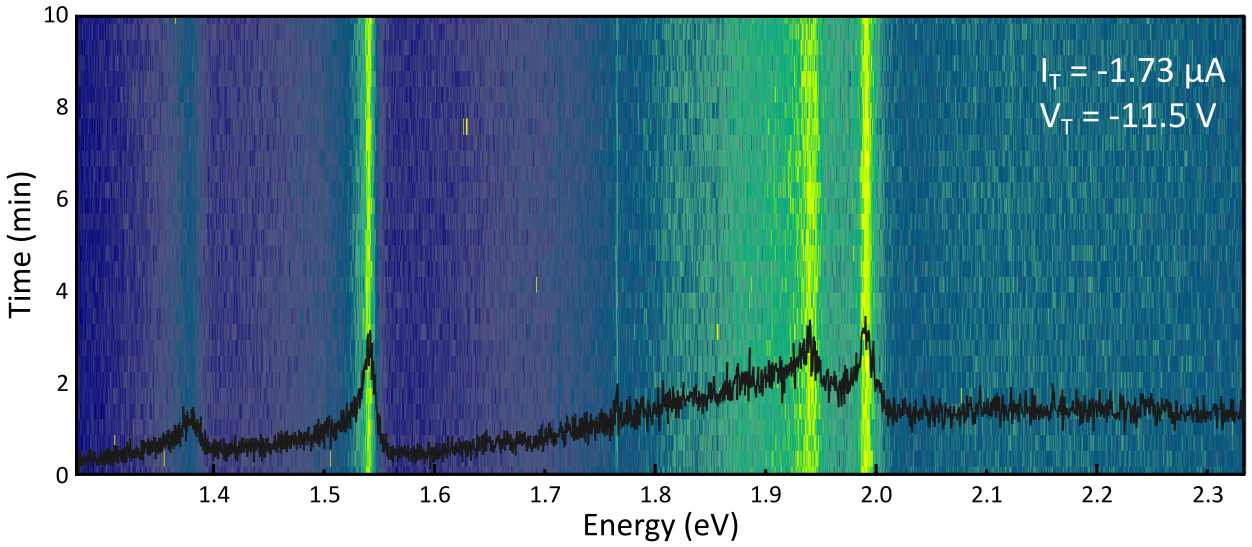

Figure S3: Time stability test of a representative sample with Gr/hBN/hBN:C/hBN/Gr structure obtained by measuring electroluminescence spectrum at 5 K from same spot for 10 minutes continuously. Sample label B05.

Temperature-dependent behavior of devices

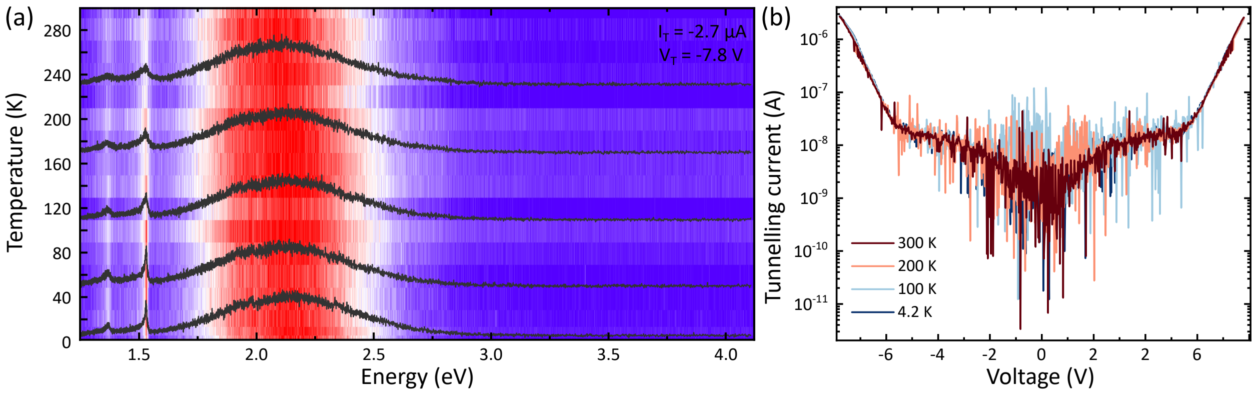

Figure S4: (a) A false-color map of the electroluminescence response, together with a few selected characteristic spectra, measured as a function of temperature in the range from 5 K up to 300 K, is presented measured with I_T = -2.7 μA. (b) Temperature dependent *IV* characteristics for the devices. Sample label N10.

We conducted comprehensive measurements over a broad temperature range of a sample without additional hBN barriers. As illustrated in Figure S4(a), our findings reveal a consistency in emission throughout the entire temperature range examined, as well as the *IV* characteristics [see Fig. S4(b)]. All the distinctive features persist in the electroluminescence

spectrum even at room temperature, underscoring the resilience of the device structure. It is worth noting that the narrow lines at energies 1.4 eV and 1.5 eV do exhibit some broadening as temperatures rise. However, this observation does not compromise the overall reliability of our devices, as they continue to deliver uniform light emission regardless of the operating conditions.

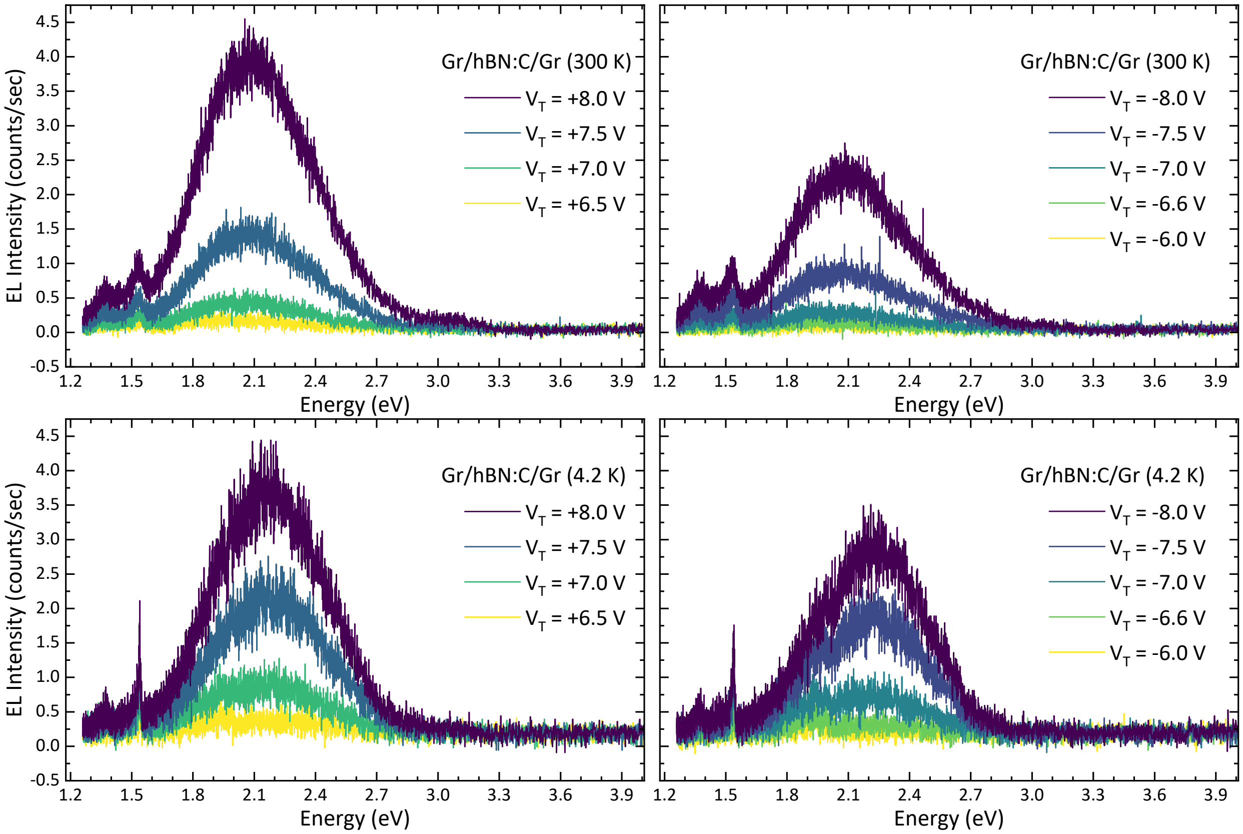

Figure S5: The evolution of the electroluminescence spectra with bias voltage under forward (a-b) and reverse (c-d) direction measured at T = 5 K (a-c) and 300 K (b-d). Sample label N10.

Additionally, we performed the voltage dependence measurements at both cryogenic temperatures (5 K) and room temperature (300 K). The data, illustrated in Figure S5, show that the overall performance of our device remains consistent. Despite the vastly different thermal conditions, the device exhibits negligible changes in its response to varying applied voltage. This demonstrates the stability and robustness of our device architecture.

Summary of all fabricated devices and comments on their performance.

	IV curve	hBN barriers	Electroluminescence	Comments
N01	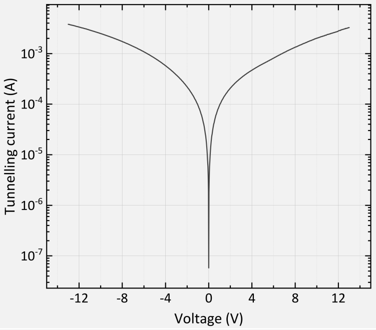	No	Yes	low resistivity, large total currents;<br>strong broad PL and EL
N02	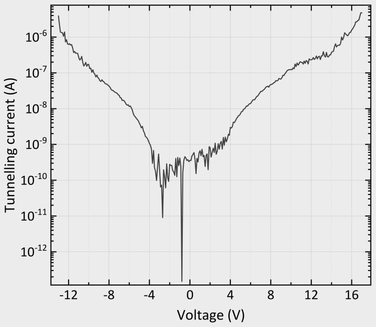	No	Yes	high resistivity, steep onset in IV curve;<br>broad PL and EL
N03	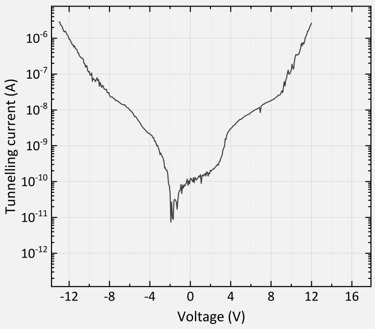	No	Yes	high resistivity, multiple steps in IV curve;<br>broad PL and EL with multiple narrow lines
N04	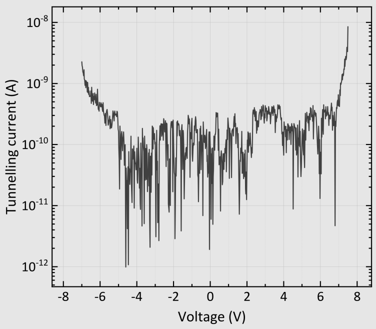	No	Yes	high resistivity, asymmetric IV curve;<br>broad, weak PL and EL

Figure S6: Current-voltage characteristics and additional information for all studied devices - part I.

	IV curve	hBN barriers	Electroluminescence	Comments
N05	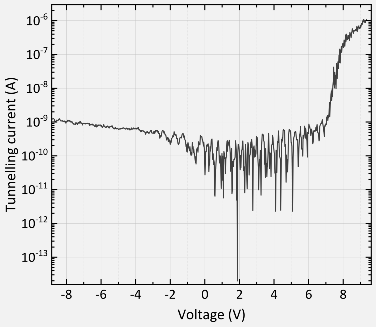	No	Yes	high resistivity, steep and asymmetric onsets in IV curve;<br>broad, weak PL and EL
N06	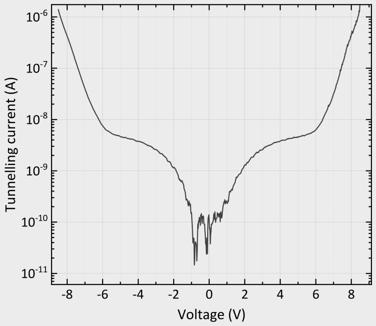	No	Yes	high resistivity, multiple steps in IV curve;<br>weak broad PL and EL
N07	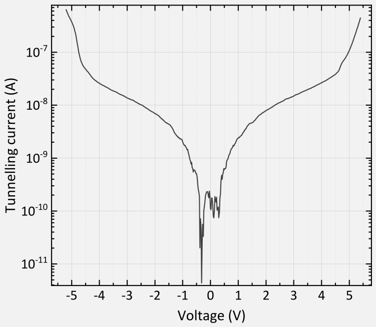	No	Yes	low resistivity, unstable IV with dielectric breakdown;<br>broad, very weak PL and EL
N08	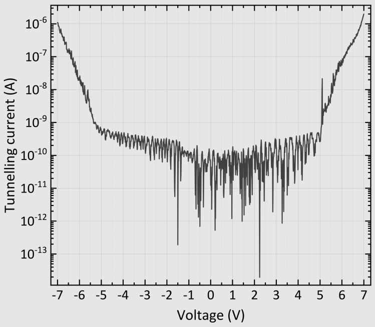	No	Yes	high resistivity, steep onset in IV curve;<br>broad PL and EL

Figure S7: Current-voltage characteristics and additional information for all studied devices - part II.

	IV curve	hBN barriers	Electroluminescence	Comments
N09	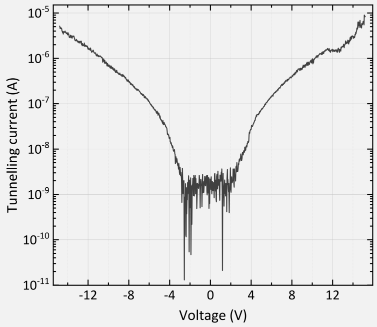	No	Yes	medium resistivity, large total currents;<br>broad PL and EL
N10	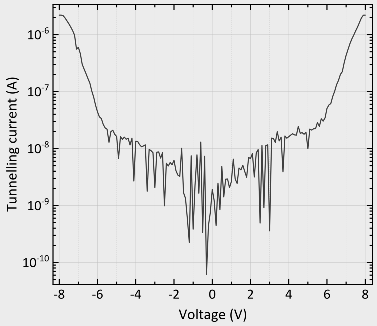	No	Yes	medium resistivity, large total currents;<br>broad PL and EL with notable narrow lines.
B01	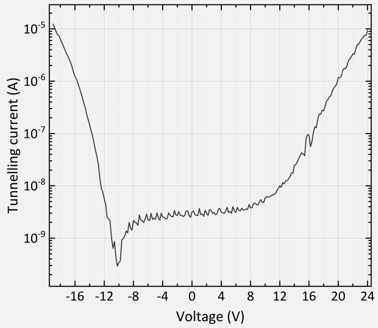	Yes	Yes	high resistivity, steep onset in IV curve, high doping level;<br>broad PL and EL with multiple narrow lines
B02	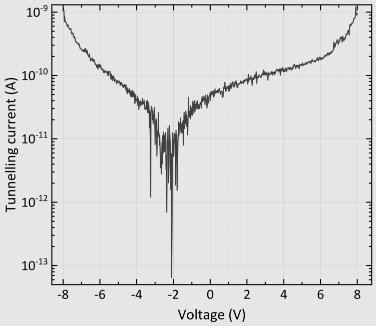	Yes	Yes	high resistivity;<br>strong broad PL and EL

Figure S8: Current-voltage characteristics and additional information for all studied devices - part III.

	IV curve	hBN barriers	Electroluminescence	Comments
B03	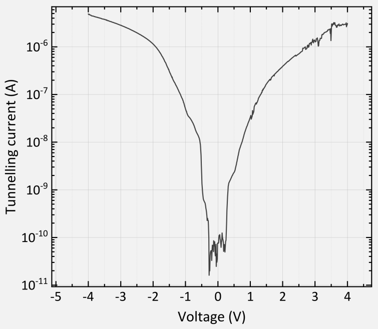	Yes	Yes	low resistivity, unstable IV with dielectric breakdown;<br>broad, very weak PL and EL
B04	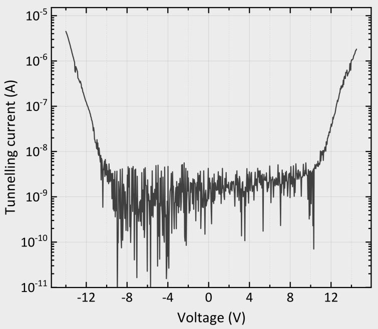	Yes	Yes	low resistivity, stable up to 140 K;<br>weak PL and EL with distinguishable narrow lines
B05	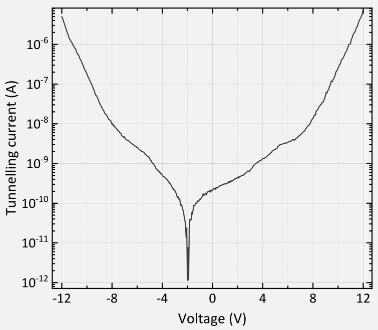	Yes	Yes	low resistivity, large total currents;<br>PL and EL with notable narrow lines

Figure S9: Current-voltage characteristics and additional information for all studied devices - part IV.

Overall 17 samples were studied (15 summarized in Figs. S6-Figs. S9 and 2 presented in the main text). Ten comprise hBN:C in between two graphene contacts (N01-N10), while the other four samples were fabricated with additional pristine hBN barriers (B01-B05). To ensure its functionality, each device was subjected to electrical testing via IV characteristics. The IV curves for all samples exhibit tunneling diode operation. However, the actual quality of the device performance was found to vary from sample to sample. A common observation was that samples with hBN barriers exhibited tunneling thresholds higher than those without, highlighting the potential impact of the structure on device performance. Although all

samples showed broad emission with varying intensity, only a few showed the presence of narrow lines, pointing to the essential role of the quality and architecture of the investigated devices.

We provide representative spectra from three different samples with and without hBN barriers, respectively. As can be seen in Fig. S10, the observation of enhancement of narrow lines on structures with additional hBN spacers is preserved. Qualitatively, the spectral line shape is comparative in different samples with a particular architecture.

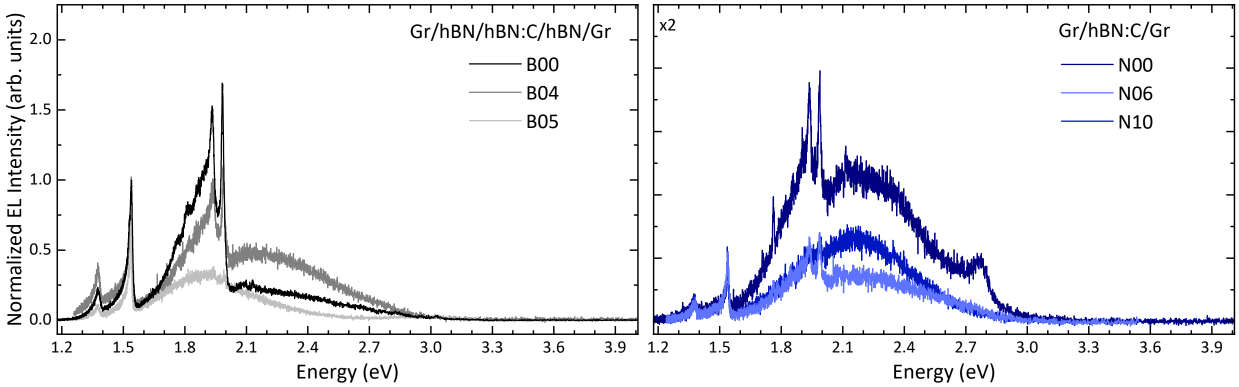

Figure S10: Normalized EL spectra from different samples with Gr/hBN/hBN:C/hBN/Gr and Gr/hBN:C/Gr architecture.

Comparison of emission efficiency in different device architectures

In Figure S11, we provide a more quantitative analysis of the electroluminescence efficiency in the samples discussed throughout the main text. Building on the qualitative differences highlighted in the previous section, we present emission spectra normalized by the current, the active area, and the acquisition time. Strikingly, our results demonstrate that the inclusion of hBN spacers leads to an 80-fold increase in emission intensity compared to samples without these barriers. This quantitative assessment underscores the pivotal role that additional hBN layers play in enhancing the electroluminescence efficiency of our devices.

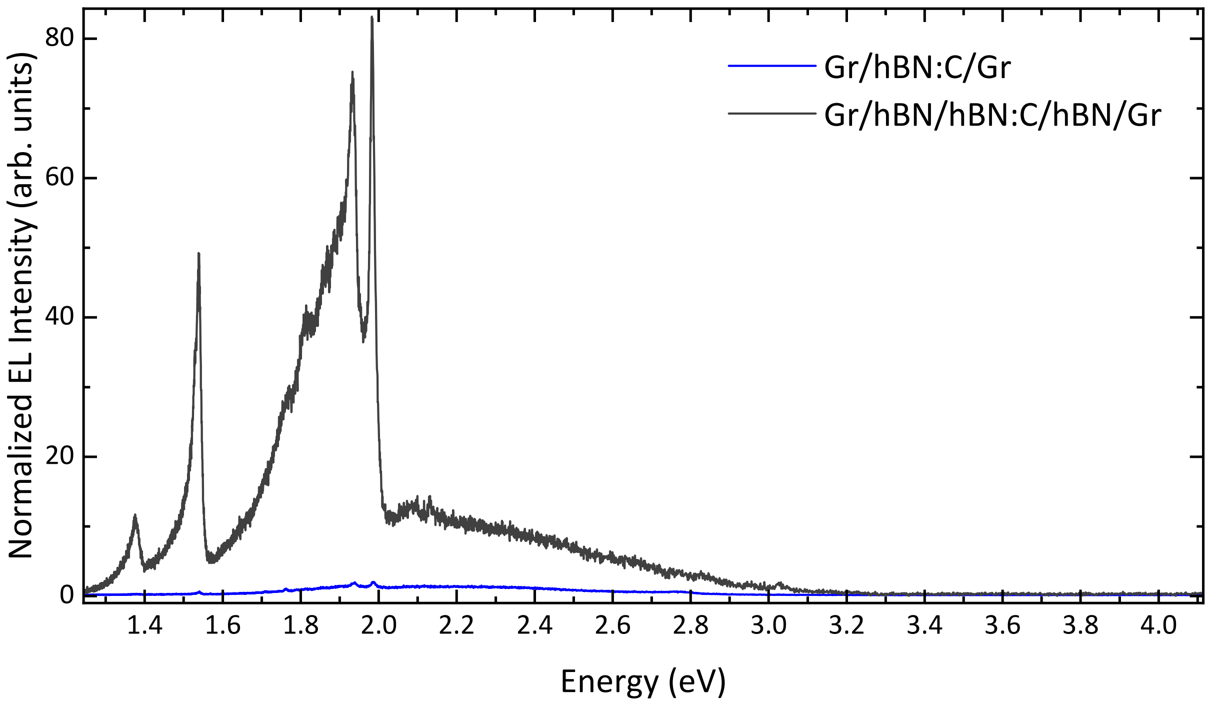

Figure S11: Electroluminescence efficiency comparison between devices with and without hBN barriers. Emission spectra from the samples discussed in the main text (sample labels N00 and B00) are normalized by both the current and active area.

Device performance after dielectric breakdown

During the course of the measurements, some of the samples underwent dielectric breakdown, in which their properties changed due to the application of high voltage. Surprisingly, some of the damaged samples still displayed broadband emission, indicating that their electroluminescent properties had not been entirely compromised. When compared with the photoluminescence of an undoped hBN sample, the emission curves exhibited similar characteristics, suggesting that during the breakdown process, the hBN:C material had been damaged, and the main recombination paths related to the defect states changed. There is a visible shift between the two curves of about 60 meV. This energy difference might be related to the dielectric environment in the studied samples.

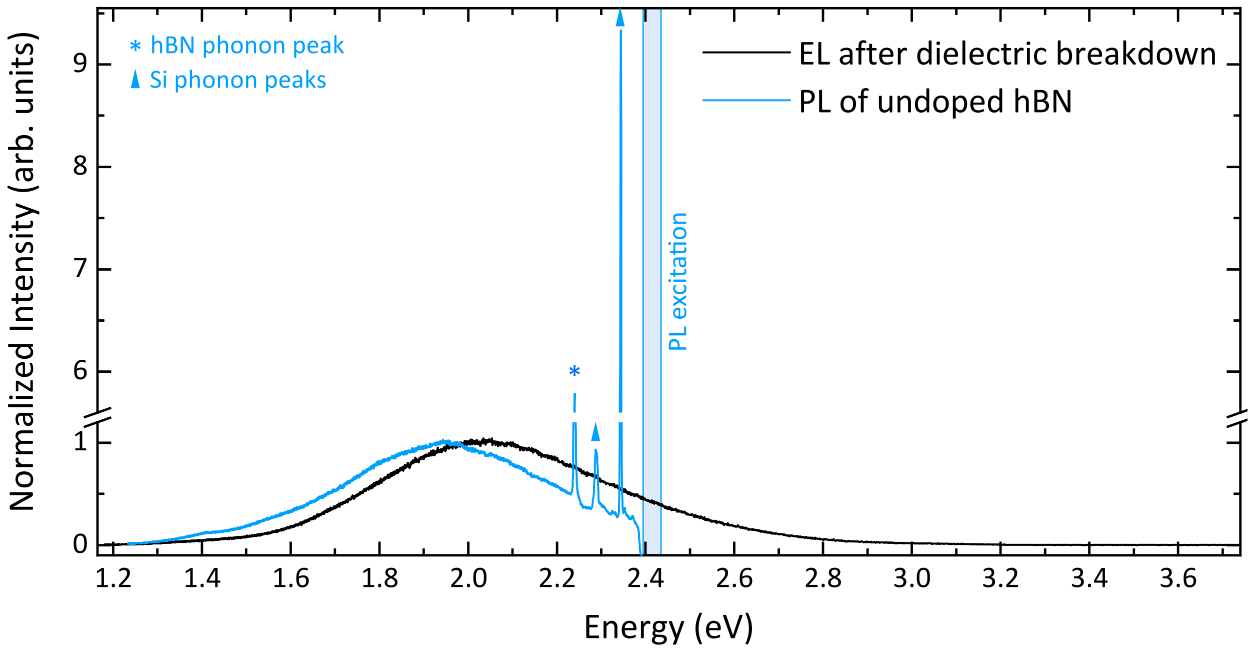

Figure S12: The comparison of the photoluminescence and electroluminescence spectra for the Gr/hBN/hBN:C/hBN/Gr device ater a dielectric breakdown. The photoluminescence spectrum was collected with 2.41 eV laser excitation. The laser was focused to a spot of 1 μm diameter with a power of 1 mW. The electroluminescence was measured with -10 V bias voltage, which corresponded to I_T = -27.4 μA. Sample label B03

Electroluminescence intensity as a function of bias voltage

In the Fig. S13, we compare the activation threshold voltage for different features observed in the emission of two different structures: Gr/hBN:C/Gr and Gr/hBN/hBN:C/hBN/Gr. For the Gr/hBN:C/Gr structure, we observe that at lower bias voltages, the narrow lines related to defects A3 and A2 can be detected, while the broadband emission only emerges for bias voltages above 5.8 V. Additionally, the electroluminescence related to the A3 line shows asymmetric charge injection and is not effective at positive bias voltages. For the Gr/hBN/hBN:C/hBN/Gr structure, this observation is even more clear, with the emergence of the A3 emission line occurring at the lowest bias voltage ($\approx$ 7.3 V). As the voltage is increased, the other defects line is activated, with the A2 line observed above 9 V, followed by the detection of both the A1 line and broadband emission for voltages above 12.5 V.

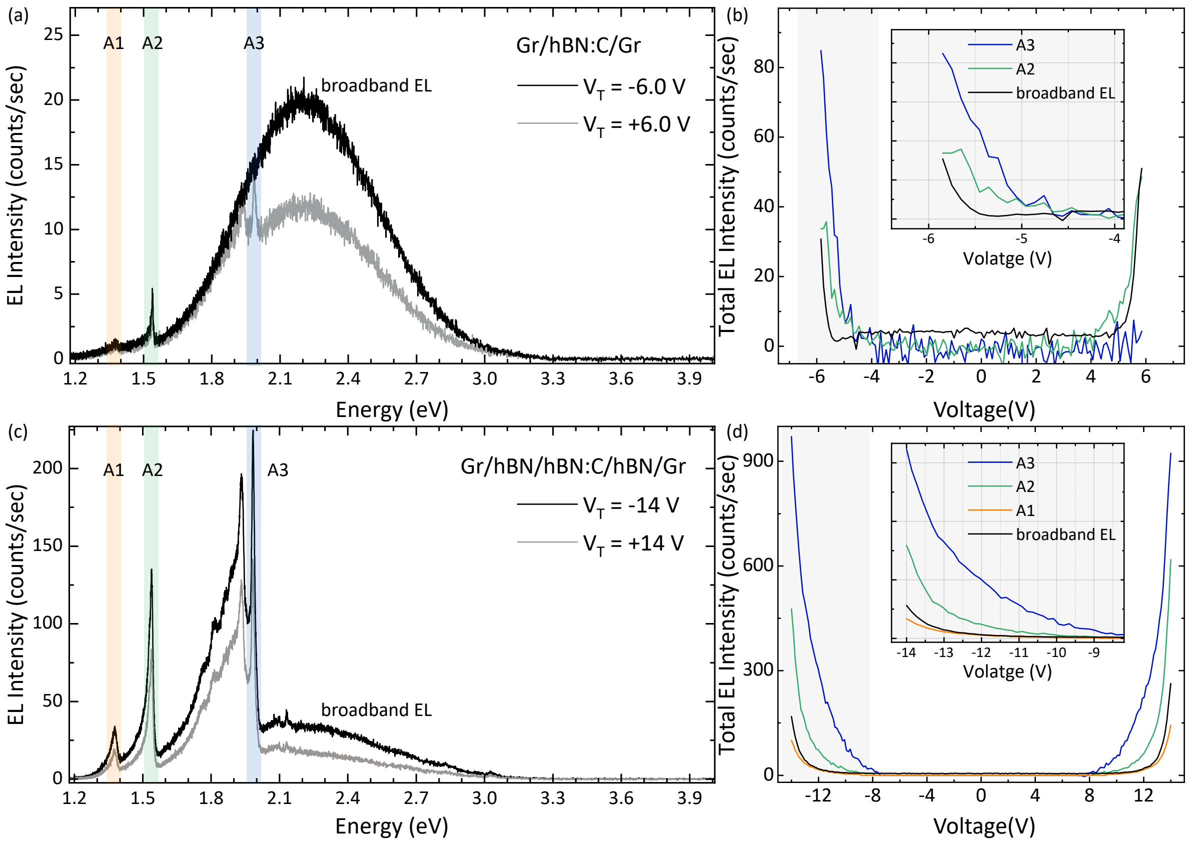

Figure S13: Electroluminescence spectra with positive and negative bias voltage (a/c) with the fitted EL intensity dependence on the tunnelling bias for selected lines (b/d) from devices without/with hBN barriers. Sample labels B00 and N00.

Defect–to–band recombination pathways

In our analysis of defect-to-band transitions in hBN:C devices, we consider three primary scenarios, as illustrated in Fig. S14. These scenarios account for the presence of both acceptor-like (AL) and donor-like (DL) states within our system. Specifically, radiative recombination occurs when the Fermi level is situated between the acceptor level and the valence band, or between the donor state and the conduction band.

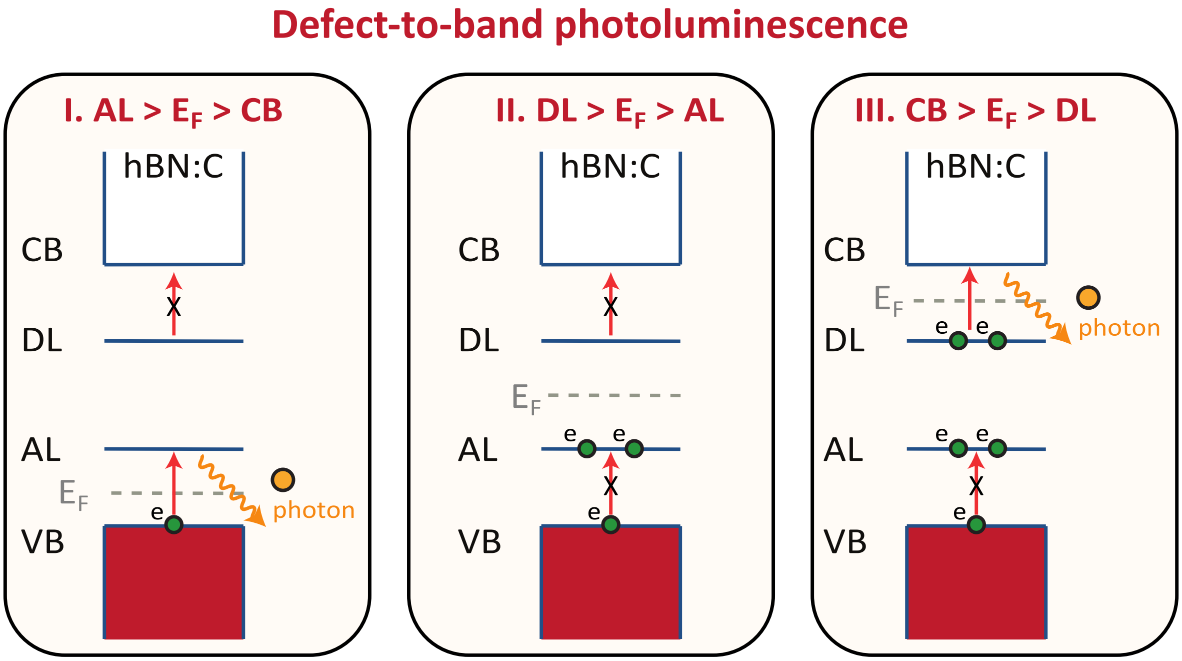

Figure S14: Schematic representation of defect-to-band transitions in hBN:C devices under different Fermi level (E_F) positions. The simplified band structure illustrates radiative recombination pathways between **(I)** the valence band (VB) and acceptor level (AL) or **(III)** donor level (DL) and conduction band (CB). These scenarios elucidate the conditions under which defect-to-band emission occurs in the device. In **(II)**, the defect-to-band recombination is not allowed due to the Fermi level being situated inbetween the defect states.

Competition between the dielectric screening and Stark effect

Figure S15 illustrates the evolution of the energy of the D2 defect as a function of applied bias voltage. We focus our discussion on this feature as it is observable for both of the studied sample architectures. However, it is worth noting that in the case of the sample with a Gr/hBN/hBN:C/hBN/Gr structure, we observe the same behavior of the emission energy for all three types of defects (A1-A3) as the tunnelling bias is increased, as discussed in the main text. For the Gr/hBN:C/Gr device, there is a clear linear dependency of the emission energy with the increased tunnelling bias throughout the considered voltage range (-5.85 – 5.85 V). This shift can be attributed to the confined Stark effect induced by the electric field, which changes the energy levels of the electrons in the system.

A more complex behavior can be seen for the sample with the Gr/hBN/hBN:C/hBN/Gr structure. Initially, the energy of the emission increases with increasing bias voltage from negative values but then decreases with further positive bias. We interpret this result as the change in charge interactions influenced by the dielectric screening of the surrounding medium. Particularly, the injection of electrons into the conduction band of hBN enhances screening, which leads to the reduction of the energy of optical transitions. The energy levels of the device are strongly affected by the dielectric screening, which can dominate the Stark effect. Such behavior is a common topic of research in quantum confined systems and is often used as a way of controlling excitonic complexes via external electrical fields.[S1,S2]

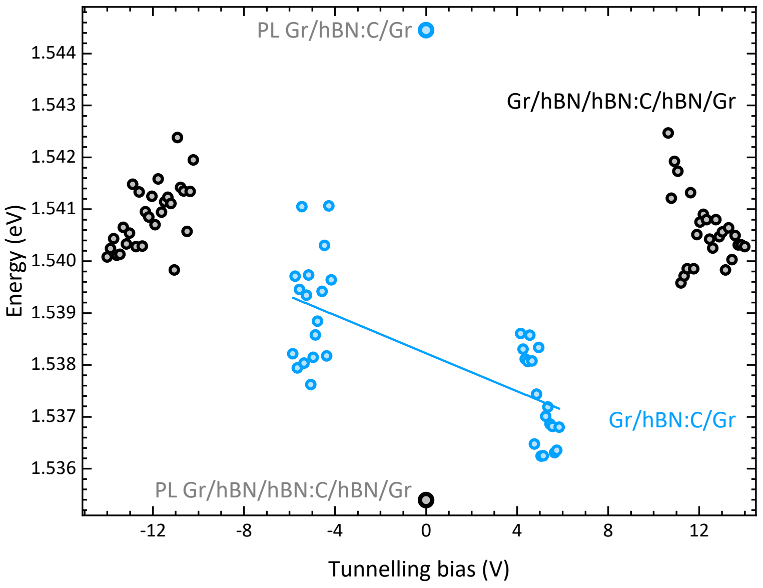

Figure S15: Peak positions of the D2 defect as a function of bias voltage for Gr/hBN:C/Gr (blue dots) and Gr/hBN/hBN:C/hBN/Gr (black dots) devices. The respective energies of the resonance in the photoluminescence spectra has been marked at 0 V. Sample labels B00 and N00.

There is also a notable difference between the energy of the peak while exciting the system electrically and optically. For Gr/hBN:C/Gr the energy shift of the peak from PL and EL spectrum equals approximately +6.2 meV, while in the case of Gr/hBN/hBN:C/hBN/Gr device the difference is -7.2 meV. Such a disparity suggests that the sample may be in different states of excitation depending on its architecture. We can hypothesize the existence of several possible excitation complexes of defect states that engage additional electrons in the ground and/or excited state. These scenarios will be outlined in the next section.

Single vs Many–body states

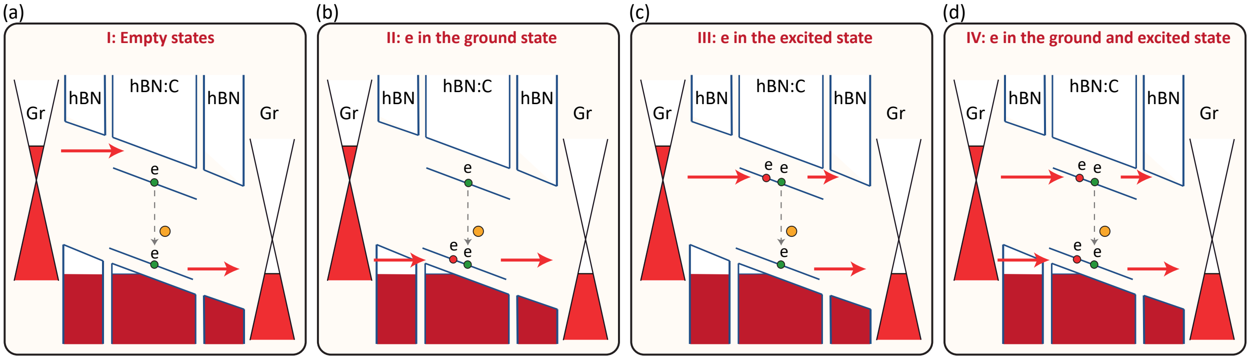

Figure S16: Schematic depiction of the additional many-body picture of charging and electroluminescence mechanism based on the band structure of the device and the Fermi level alignment with bias (a) considering an additional electron in the ground state (b), excited state (c) or both (d).

We propose alternative many-body processes that can explain observed defect transitions in our electroluminescence results. Specifically, we propose that additional electrons may be residing in the ground, excited, or both states, leading to these observed defect transitions and their particular behavior in different tunneling regimes. When additional electrons are involved, the exchange interactions between the electrons can affect the transition energies yielding the onset of electroluminescence. At high bias, the system can be in the ground electronic state, with an electron injected into the conduction band that relaxes to the underlying defect level. Similarly, a hole can be injected into the valence band, enabling the recombination of an electron occupying a defect level. Additionally, an electron can tunnel through an empty hBN band gap to the defect level in hBN:C. The specific electronic structure of the mid-gap levels, in combination with the position of the Fermi level in graphene relative to the hBN band edges forming the triangular tunnel barrier, determines whether an electron is inserted into the ground or excited defect state. This electron may further tunnel to the graphene layer, completing the charge transfer process. Overall, the behavior of the system under high bias is complex and depends on several factors, including the electronic properties of the defect levels and the energy barriers associated with tunneling through the

hBN:C and graphene layers. Further investigation is necessary to fully understand these many-body processes and their contributions to the behavior of the system under different tunnelling regimes.

References

[S1] Finley, J.; Sabathil, M.; Vogl, P.; Abstreiter, G.; Oulton, R.; Tartakovskii, A.; Mowbray, D.; Skolnick, M.; Liew, S.; Cullis, A., et al. Quantum-confined Stark shifts of charged exciton complexes in quantum dots. *Physical Review B* **2004**, *70*, 201308.

[S2] Abraham, N.; Watanabe, K.; Taniguchi, T.; Majumdar, K. Anomalous Stark shift of excitonic complexes in monolayer WS_2. *Physical Review B* **2021**, *103*, 075430.
